# Impact of Loading Phase, Initial Response and *CFH* Genotype on the Long-Term Outcome of Treatment for Neovascular Age-Related Macular Degeneration

**DOI:** 10.1371/journal.pone.0042014

**Published:** 2012-07-25

**Authors:** Moreno Menghini, Barbara Kloeckener-Gruissem, Johannes Fleischhauer, Malaika M. Kurz-Levin, Florian K. P. Sutter, Wolfgang Berger, Daniel Barthelmes

**Affiliations:** 1 Department of Ophthalmology, University Hospital of Zurich, Zurich, Switzerland; 2 Institute of Medical Molecular Genetics, University of Zurich, Schwerzenbach, Switzerland; 3 ETH Zurich, Institute of Biology, Zurich, Switzerland; 4 Neuroscience Center Zurich (ZNZ), University of Zurich, Zurich, Switzerland; 5 Center for Integrative Human Physiology (ZIHP), University of Zurich, Zurich, Switzerland; 6 Save Sight Institute, University of Sydney, Sydney, Australia; Innsbruck Medical University, Austria

## Abstract

**Objective:**

Factors influencing the outcome of anti-VEGF treatment in neovascular AMD are still investigated. We analyzed the impact of a loading phase, the significance of an initial response for the long-term and the effect of the *CFH* polymorphism (p.His402Tyr) on treatment outcome.

**Methods:**

Patients treated with ranibizumab for neovascular AMD were analyzed over a period of 24 months by assessing effects of loading phase, initial response and genotype of *CFH* rs1061170 (c.1204C>T, p.His402Tyr).

**Results:**

204 eyes were included. A change of +5.0 [−1;+11] letters and +1.5 [−5.5;+9.5] was observed with a median of 4 [3]; [7] and 10 [7]; [14] ranibizumab injections during 12 and 24 months, respectively. Loading phase was no significant predictor for treatment as VA outcome in eyes with and without loading phase was similar (p = 0.846 and p = 0.729) at 12 and 24 months. In contrast, initial response was a significant predictor for improving vision of 5 or more letters at 12 (p = 0.001; OR = 6.75) and 24 months (p = 0.01; OR = 4.66). Furthermore, the CT genotype at *CFH* rs1061170 was identified as a significant predictor for a favorable VA outcome at 12 and 24 months (OR = 6.75, p = 0.001 and OR = 4.66, p = 0.01).

**Conclusions:**

Our data suggest that clinical decisions regarding treatment may be guided by observing patients’ initial response as well as their genotype of SNP rs1061170, while the criterion of loading phase may not bear the customary value.

## Introduction

Age-related macular degeneration (AMD) is the leading cause of severe visual impairment in the elderly population in developed countries [1]. For example, in Australia where AMD is the leading cause of blindness [2]. AMD affects 1 in 7 Australians over the age of 50 years with increasing incidence with age [3]. Overall, 17700 new patients per year are diagnosed having AMD [4]. Advanced stages present either as geographic atrophy or choroidal neovascularization [5]. Anti-vascular-endothelial growth-factor (VEGF) antibodies have revolutionized the treatment of neovascular AMD. The most widely used compounds are ranibizumab (Lucentis®, Novartis, Basel, Switzerland; Genentech Inc., South San Francisco, CA, USA) and bevacizumab (Avastin®, Roche, Basel, Switzerland; Genentech Inc., South San Francisco, CA, USA). In January 2007, ranibizumab was approved by the FDA for neovascular AMD after having shown to improve visual acuity with monthly injections in patients with neovascular AMD in the MARINA [6] and ANCHOR [7] studies and has now become the standard treatment for neovascular AMD.

The study regimen of monthly injections, as applied in MARINA and ANCHOR, can hardly be realized in a typical clinical setting as a monthly schedule results in a heavy burden of treatment for both the patient and family members that often accompany elderly and visually impaired patients to the clinic visits. Given the high number of patients affected, manageability of high patient numbers requiring potentially a lifelong treatment has become a significant issue. Therefore further studies have been conducted to identify treatment schedules that achieve successful treatment results with a lower number of injections [8]–[12]. Widely applied is a so-called loading phase with three initial consecutive intravitreal injections of ranibizumab followed by a PRN scheme (pro re nata) [9], [10], [13]. To date, there is no convincing evidence though to support the idea that a loading phase is superior to an immediate PRN scheme, where the re-treatment regimen is guided by both morphological and clinical parameters [14], [15]. Since there is a substantial number of patients presenting an unfavorable long-term outcome, i.e., loss of vision despite continued treatment [16], factors other than treatment regimen possibly influencing treatment responses are scrutinized and indicators for response to treatment are evaluated.

The initial response to anti-VEGF therapy was suggested to be indicative for the long-term response, but available data so far are contradicting [16]–[18].

On the other hand, while the influence of genetic risk factors on AMD development is well established [19]–[23], there is now evidence showing that genetic variants also influence treatment results and seem responsible for the variation in outcome despite similar treatment patterns [24]–[29]. Specifically, we have previously shown that the CC genotype at *CFH* rs1061170 is associated with an unfavorable visual acuity course after 12 months of ranibizumab therapy [24]. Despite the progress made, a better understanding of the impact of different factors on the treatment outcome is still somewhat unclear, partially due to the fact that hypotheses are tested in different study populations and, as a consequence, results are not always comparable. Additionally, many studies had rather short-term endpoints, i.e. 6 months or 12 months of follow-up only. The work presented here offers the advantage of analyzing a single study population over an extended period of 24 months. It addresses the following questions: (i) Does a loading phase have an influence on the long-term treatment outcome? (ii) Is an initial response predictive of a favorable long-term outcome, i.e., sustained gain of vision? and (iii) Are genetic predictors accountable not only after 12 months of treatment but also for a long-term treatment of 24 months?

## Methods

This is a retrospective analysis of patients treated for neovascular AMD with ranibizumab at the Department of Ophthalmology at the University Hospital Zurich, Switzerland. The treatment regimen was physician-guided, patients were not assigned to a predefined schedule. Patients with prior treatments or treatments other than intravitreal ranibizumab were excluded. A detailed description of the clinical data collection as well as DNA preparation and genetic analysis for this study was published previously [24]. Briefly, visual acuity was recorded using logMAR charts. Patients were treated with 0.5 mg ranibizumab, administered intravitreally. Subsequent injections were only performed if signs of lesion activity (i.e. subretinal fluid, cystoid macular edema, sub- or intraretinal bleeding, active lesion in fluorescein angiography) were present. Follow up visits were scheduled monthly, but could vary due to patients’ personal needs. The study was conducted according to the Tenets of the Declaration of Helsinki and approved by the local ethics committee.

Two response groups at 12 and 24 months after start of treatment were established: good responders (GR) containing eyes that had a 5 or more letter improvement compared to baseline and poor responders (PR) comprising of eyes losing 5 or more letters compared to baseline.

An initial loading phase was defined as the administration of three consecutive intravitreal injections of ranibizumab in 4 weekly intervals at the start of the therapy, i.e. at baseline, 1 month and 2 months after baseline. To assess the effect of the loading phase, eyes were allocated to two groups: those that had received a loading phase and those that did not. The course of VA at 12 and 24 months was compared between the two groups, i.e., loading phase vs. no loading phase, as well as clinical characteristics (baseline VA, age, gender, injection frequency and visits) and the distribution of the genotype of *CFH* rs1061170. The distribution of eyes with and without loading phase to GR and PR was analyzed.

An initial response to treatment was defined as an improvement in visual acuity (VA) of 5 or more letters compared to the baseline VA at 1, 2 and 3 months after commencing treatment.

Similar to the first analysis, the course of VA at 12 and 24 months was compared between the two groups, i.e. initial response vs. no initial response, as well as the distribution of eyes with and without initial response to GR and PR.

Genetic analysis was performed for the SNP *CFH* rs1061170 as described [24]. Based on the genotype of *CFH* rs1061170, the course of VA at 12 and 24 months was compared between groups with respect to clinical characteristics (baseline VA, age, gender, loading phase, injection frequency and visits) at 12 and 24 months in GR and PR and the distribution of the *CFH* genotype at rs1061170 were assessed.

**Table 1:** Effects of genotype SNP rs1061170 at *CFH*.

		Genotype		
		CC	CT	TT
		N	N	N
change ≥5 month 12	loss ≥5 letters	17	16	11
	gain ≥5 letters	15	46	22
change ≥5 month 24	loss ≥5 letters	17	14	12
	gain ≥5 letters	10	28	17
Percentilesmonth 12	25	20	19	16
	75	7	30	14
Percentilesmonths 24	25	14	12	12
	75	5	22	14

Frequencies of genotypes are given for the groups with gain or loss of ≥5 letters as well as the upper and lower percentiles at 12 and 24 months after treatment begin.

### Statistical Analysis

Statistical calculations were done using either commercially available software packages (IBM SPSS Statistics 19, SPSS Inc. Chicago, Il or SAS software, SAS Institute Inc., NC, USA) or open access internet portals from http://in-silico.net/statistics. Normally distributed data are presented as mean±standard deviation (SD), not-normal data are presented as median and interquartile range [IQR]. Normality was assessed using the Shapiro-Wilks test. Odds ratios from independent samples were compared as described [24]. Odds ratios from 2×2 and 2×3 tables were analyzed using Fisher’s exact test.

Generalized estimation equation (GEE) methods were used (binary outcome) in three separate models to analyze the predictive value of loading phase, initial response and *CFH* rs1061170 and to address the fact that in some patients both eyes were included. Additional predictors were gender, age at baseline, VA at baseline and lesion type.

The dependent variable (GR and PR at 12 and 24 months) was coded binary, PR being the reference category. For binary predictors the reference categories were: no loading phase, no initial response, CC genotype and male gender. For the categorical predictor lesion type, minimally classic was chosen as reference category. VA at baseline and age at baseline were entered as continuous variables. Since no difference in number of injections, number of visits or injections/months could be identified for any of the groups analyzed, number of injections and visits were not included in the GEE modeling.

## Results

### Characteristics of the Study Population

**Figure 1:**
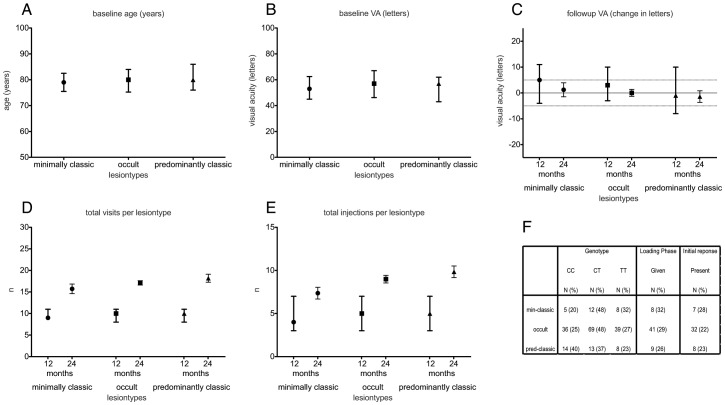
Overview of three different lesion-types. Values are displayed for age at baseline (A), visual acuity at baseline (B), VA at 12 and 24 months (C), number of visits in 12 and 24 months (D), total injections in 12 and 24 months (E) and the distribution of genotypes (F). Symbols show the median while whiskers indicate the interquartile range. No statistically significant differences are found.

Clinical and genotyping data were collected for 204 eyes. In 10 patients, both eyes were included in the analysis. 63.2% of the patients were female. The average age in all patients at baseline was 79.3±7.1 years. The mean baseline visual acuity in all eyes (VA) was 55.2±14.6 letters. 70.5% of all eyes showed an occult lesion type at baseline, 12.3% had minimally classic and 17.2% had predominantly classic lesions. The median number of ranibizumab injections in all eyes after 12 and 24 months was 4 [3]; [7] and 10 [7]; [14], respectively. The median number of follow up visits in all eyes was 9 [9]; [11] after 12 months and 20.5 [18]; [23] over the period of 24 months. In all eyes, a change in VA of +5.0 [−1;+11] letters was achieved at 12 months and +1.5 [−5.5;+9.5] letter 24 months from baseline. No statistically significant differences were found between the three different lesion types (occult, minimally classic, predominantly classic) with respect to age (Fig. 1A), baseline VA (Fig. 1B), VA course (Fig. 1C), number of visits and injections (Fig. 1D and E), *CFH* genotype, eyes with loading phase, and eyes with initial response (Fig. 1F). Analysis of the distribution of the SNP rs1061170 at *CFH* showed that 46.1% (N = 94) of all patients carried the CT genotype, while 27% (N = 55) had the CC and 27% (N = 55) the TT genotype. Of the 204 eyes, 83 eyes (40.6%) showed a change of 5 or more letters after 12 months. 77 eyes (37.7%) experienced a loss of 5 or more letters after 12 months. After 24 months 55 eyes (37.6%) of 146 eyes gained 5 or more letters, while 48 eyes (32.8%) lost 5 or more letters. In 58 eyes no VA data was available at 24 months after baseline.

### Loading Phase

**Figure 2:**
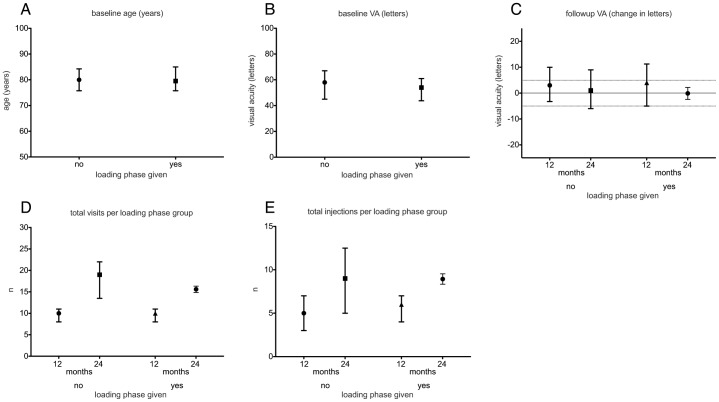
Overview of the loading phase groups. Values are displayed for age at baseline (A), visual acuity at baseline (B), VA at 12 and 24 months (C), number of visits in 12 and 24 months (D), total injections in 12 and 24 months (E) and distribution of CFH genotypes (F) in the loading phase groups. Symbols show the median while whiskers indicate the interquartile range. No statistically significant differences are found.

Of the 204 eyes in the study, 58 (28.4%) initially received three consecutive intravitreal injections of ranibizumab (loading phase). No statistically significant differences were found with respect to age (Fig. 2A), baseline VA (Fig. 2B), number of visits and number of injections between eyes with and without loading phase (Fig. 2D and E). The distribution of the *CFH* polymorphism between the two groups was comparable (P = 0.72, Fig. 2F). VA at 12 and 24 months after baseline did not show significant differences (p = 0.846 and p = 0.729, Fig. 2C). GEE analysis did not identify loading phase to be significant predictor with respect to GR/PR at 12 and 24 months (p = 0.881 and p = 0.472, respectively).

### Initial Response

**Figure 3:**
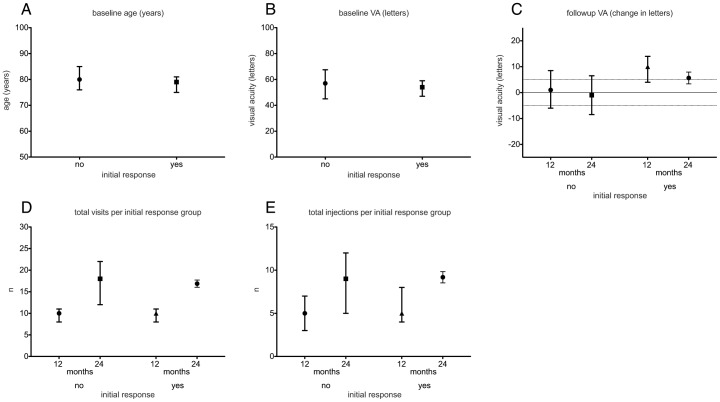
Overview of the initial response groups. Values are displayed for age at baseline (A), visual acuity at baseline (B), VA at 12 and 24 months (C), number of visits in 12 and 24 months (D), total injections in 12 and 24 months (E) and distribution of CFH genotypes (F) in the initial response groups. Symbols show the median while whiskers indicate the interquartile range. No statistically significant differences are found.

An initial improvement of 5 or more letters at 1, 2 and 3 months after commencement of treatment (initial response) was achieved in 47 eyes (23%). 16 (34%) of these also belonged to the group of eyes that had received a loading phase. No statistically significant difference was found in the proportions of eyes showing an initial gain between eyes that had received a loading phase and those that did not (p = 0.3591). No statistically significant differences were found for age (Fig. 3A), baseline VA (Fig. 3B), VA course (Fig. 3C), number of visits and number of injections (Fig. 3D and E). Of the 47 eyes with an initial response to treatment, 74.5% maintained the improvement of 5 or more letters at 12 months and 66.6% maintained the initial response until 24 months. GEE analysis revealed that an initial response is a significant predictor for improving vision of 5 or more letters at 12 (p = 0.001; OR = 6.75, CI 2.17–20.98) and 24 months after baseline (p = 0.01; OR = 4.66, CI 1.44–15.03). Importantly, of the 157 eyes that did not show an initial response, 30.6% showed an increase of 5 or more letters 12 months from baseline and 29.2% at 24 months from baseline. The distribution of the genotypes for *CFH* polymorphism rs1061170 was not significantly different between eyes that showed an initial gain and those that did not (p = 0.5133, Fig. 3F).

### CFH Genotype

The distribution of the possible genotypes at *CFH* rs1061170 between the two groups GR and PR (≥5 letters vs. ≤5 letters change compared to baseline) showed statistically significant differences both at 12 and 24 months after baseline (Table 1). The CC genotype at *CFH* rs1061170 (homozygous for His at protein position 402 in CFH) was found at a statistically significant higher frequency in the PR group at 12 and 24 months (p = 0.0174 and p = 0.0236). GEE analysis revealed that the CT genotype is a significant predictor for a favorable VA outcome both at 12 and 24 months (OR = 6.75, p = 0.001 and OR = 4.66, p = 0.01, respectively). No statistically significant differences between the three genotype groups were found for gender, age at baseline, lesion type, loading phase, initial response and baseline VA. For comparison with our previously published data [24] we also assessed treatment response by defining good and poor responders according to the grouping of percentiles (75^th^ percentile and 25^th^ percentile; Table 1). With this higher discriminatory power the differences in the genotype distribution were even more pronounced. The statistical significance level for the CC genotype and poor response was P = 0.0082 at 12 months and P = 0.0167 at 24 months (Table 2).

**Table 2:** Effects of genotype SNP rs1061170 at *CFH*.

	genotype	OR	LL	UL	Fisher’s Exact	
change ≥5 month 12	CC vs. CT	3.26	1.33	7.99	0.0121	0.0326
	CC vs. TT	2.27	0.83	6.18	0.1361	
	CC vs. CT/TT	2.85	1.25	6.52	0.0174	
change ≥5 month 24	CC vs. CT	3.40	1.24	9.35	0.0251	0.0486
	CC vs. TT	2.41	0.82	7.06	0.1194	
	CC vs. CT/TT	2.94	1.17	7.37	0.0236	
percentiles month 12	CC vs. CT	4.51	1.60	12.70	0.0041	0.0123
	CC vs. TT	2.50	0.82	7.67	0.1687	
	CC vs. CT/TT	3.59	1.36	9.46	0.0082	
percentiles months 24	CC vs. CT	5.13	1.49	17.74	0.0103	0.0271
	CC vs. TT	3.27	0.91	11.75	0.0772	
	CC vs. CT/TT	4.20	1.34	13.19	0.0167	

Statistical assessment of the frequencies of the genotypes for the groups with gain or loss of ≥5 letters as well as the upper and lower percentiles at 12 and 24 months after treatment begin. OR: odds ratio; LL and UL: lower and upper limit.

Interaction effects of loading phase and *CFH* genotype as well as the initial response and *CFH* genotype could not be identified.

## Discussion

We were able to identify two robust significant predictors for a favorable long-term outcome in neovascular AMD treatment with ranibizumab. The *CFH* genotype as well as the initial response both shows statistically significantly association with a good long-term VA course. The effects of the *CFH* genotype and the initial response seem to be independent, i.e. a patient carrying the CT genotype at *CFH* rs1061170 and showing an initial response does not double the likelihood of experiencing a good VA course over the long term in comparison with a patient who “only” carries either variable. The loading phase, on the other hand, does not seem to positively influence the VA in the long term.

In nowadays-busy clinical settings, monthly injections of ranibizumab are hardly feasible due to increasing numbers of patients amenable for treatment and the high workload associated to it. Consequently, different therapeutic regimens were elaborated and have been tested. The most consistent strategy applied by clinicians is the initial loading phase consisting of three consecutive intravitreal injections. This regimen was first introduced by the PrONTO study [9]. The evidence available from many phase III clinical trials (e.g. PIER [10], SAILOR [30], SUSTAIN [17] and EXCITE [18]) has been assessed by an international retina expert panel suggesting a superiority of an initial loading phase followed by a monthly monitoring [11]. However, these studies always evaluated a flexible strategy after the initiation phase. To our knowledge there has not been a study assessing the efficacy of the loading phase with respect to the long-term response. Our analysis did not reveal a beneficial outcome for patients who received a loading phase compared to those who did not receive one. This finding is important because it indicates that perhaps many AMD patients do not necessarily need three consecutive initial anti-VEGF injections in order to achieve a long-term success. The OCT and clinical findings guided administration of anti-VEGF from the beginning seems to be sufficient in distinguishing between patients who continue to show signs of lesion activity after the first injection and hence need repeated injections and those patients who are “dry” after less than three injections.

The initial response, on the other hand, is of high prognostic value. In the MARINA study patients with an initial response (defined as VA change >0) gained 13 letters logMAR after 12 months compared to the loss of 3 letters logMAR of initial non-responders (VA change <0) [6]. The PIER trial showed that a smaller proportion of initial gainers (40%) maintain their initial gain after 12 months, but all the remaining initial gainer still fare better than patients with no initial gain (defined as VA change >0) [10]. In our study, we defined the initial gain as increase of 5 or more letters logMAR at 1, 2 and 3 months from baseline. With this definition only 23% of the eyes fulfilled the criteria, but approximately 75% of these eyes maintained the increase of 5 or more letters logMAR over a period of 12 months and 67% maintained the initial response until 24 months. More importantly, of the remaining eyes that did not experience an initial gain, 31% showed an increase of 5 or more letters after 12 and 29% after 24 months from baseline. In other words: Of all eyes that gained 5 or more letters after 12 and 24 months, 58% and 60% had not shown an initial gain and yet did benefit from continued therapy.

These findings are of high value for every ophthalmologist confronted with questions of discussing a patient’s long-term VA course, because the patient with an initial gain can be reassured that his/her odds for a good course are positive and on the other hand the patient with no initial gain can be encouraged that a realistic probability does exist that she/he might be a long-term gainer because the lack of an initial response does not exclude a chance for improvement. Hence it is clinically important to evaluate the initial response in each patient undergoing treatment for wet AMD.

The third question we addressed concerned the genetic influence on the long-term response to treatment. As previously published, the *CFH* polymorphism rs1061170 (CC genotype, homozygous for the His allele of CFH at position 402) seems to be associated with a poor response to ranibizumab after 12 months [24]. In the current analysis, we wanted to test whether these findings are also true for the response after 24 months. The CT genotype (heterozygous for the His and Tyr alleles at position 402 of CFH) was associated with an odds ratio of 3.2 and of 3.6 for a VA increase of 5 or more letters after 12 and 24 months, respectively. These results strengthen the impact of genetic variations on the response to treatment. Possibly, variations in the *CFH* gene influence the response to treatment through modifying VEGF levels and expression of additional angiogenic and inflammatory factors [31]–[33]. Alternatively, genetic variations might also account for a different AMD “phenotype” with a different composition of the lesion less susceptible to the approved therapies [34]. These hypotheses though warrant further research in the pathophysiology of AMD and the impact by the genetic background.

The fact that in the study population the effect of *CFH* and initial response on the final outcome seem to occur independently may be due to several reasons. Patients with an initial increase may have e.g. less aggressive lesions or a rather minor amount of morphological damage whereas vision impairment could be mainly caused by retinal edema and to a lesser extent by neuroretinal damage, thus explaining the quick initial improvement. However, further investigations of retinal pathology are necessary to address these questions. As mentioned, the *CFH* genotype may act via inflammatory or angiogenic pathways. This may lead to a beneficial long-term effect contributing to the majority of eyes eventually achieving 5 or more letters at 12 and 24 months. It may hence be hypothesized that patients with less aggressive lesions benefit from ranibizumab treatment due to quick edema resolution resulting in a rapid initial improvement that is sustained in the majority of cases while patients with *CFH* CT genotype profit in the long term especially from a combination of repetitive treatment and the “advantageous” genotype by as of yet unclear interactions of gene expression changes and lesion development.

Taken together, we could not find a clear benefit of the loading phase scheme regarding the long-term VA outcome. The initial response however is an important predictor for a good long-term VA course. The converse argument is not true, since initial poor responders may still experience a favorable long-term VA course. Furthermore, we were able to confirm the most consistently found AMD susceptibility variant, the *CFH* polymorphism, to account for different responses in treatment even after 24 months. Patients carrying the CT genotype at *CFH* rs1061170 have an approximately three times higher probability to experience a clinically significant long-term gain in visual acuity with a PRN ranibizumab scheme.
